# Proteomic characterization and discrimination of *Aeromonas* species recovered from meat and water samples with a spotlight on the antimicrobial resistance of *Aeromonas hydrophila*

**DOI:** 10.1002/mbo3.782

**Published:** 2019-01-06

**Authors:** Ayman Elbehiry, Eman Marzouk, Eman Abdeen, Musaad Al‐Dubaib, Abdullah Alsayeqh, Mai Ibrahem, Mohamed Hamada, Afrah Alenzi, Ihab Moussa, Hassan A. Hemeg

**Affiliations:** ^1^ Department of Bacteriology, Mycology and Immunology, Faculty of Veterinary Medicine University of Sadat City Sadat City Egypt; ^2^ Department of Public Health, College of Public Health and Health Informatics Qassim University Buraidah Saudi Arabia; ^3^ Department of Medical laboratories, College of Applied Medical Science Qassim University Buraidah Saudi Arabia; ^4^ Department of Veterinary Medicine, College of Agriculture and Veterinary Medicine Qassim University Buraidah Saudi Arabia; ^5^ Department of Public Health, College of Applied Medical Science King Khalid University Abha Saudi Arabia; ^6^ Department of Fish Diseases and Management, Faculty of Veterinary Medicine Cairo University Cairo Egypt; ^7^ Department of Food Hygiene & Control, Faculty of Veterinary Medicine University of Sadat City Sadat City Egypt; ^8^ Department of Botany and Microbiology, College of Science King Saud University Riyadh Saudi Arabia; ^9^ Department of Microbiology, Faculty of Veterinary Medicine Cairo University Cairo Egypt; ^10^ Department of Medical Technology/Microbiology, College of Applied Medical Sciences Taibah University Madinah Saudi Arabia

**Keywords:** *Aeromonas* spp., antimicrobial resistance, differentiation, microchannel electrophoresis, protein fingerprinting

## Abstract

*Aeromonas* is recognized as a human pathogen following ingestion of contaminated food and water. One major problem in *Aeromonas* identification is that certain species are phenotypically very similar. The antimicrobial resistance is another significant challenge worldwide. We therefore aimed to use mass spectrometry technology for identification and discrimination of *Aeromonas* species and to screen the antimicrobial resistance of *Aeromonas hydrophila (A. hydrophila)*. A total of 150 chicken meat and water samples were cultured, and then, the isolates were identified biochemically by the Vitek^®^ 2 Compact system. Proteomic identification was performed by MALDI‐TOF MS and confirmed by a microchannel fluidics electrophoresis assay. Principal component analysis (PCA) and single‐peak analysis created by MALDI were also used to discriminate the *Aeromonas* species. The antimicrobial resistance of the *A. hydrophila* isolates was determined by Vitek^®^ 2 AST cards. In total, 43 samples were positive for *Aeromonas* and comprised 22 *A. hydrophila*, 12 *Aeromonas caviae* (*A. caviae*), and 9 *Aeromonas sobria* (*A. sobria*) isolates. Thirty‐nine out of 43 (90.69%) *Aeromonas* isolates were identified by the Vitek^®^ 2 Compact system, whereas 100% of the *Aeromonas* isolates were correctly identified by MALDI‐TOF MS with a score value ≥2.00. PCA successfully separated *A. hydrophila*,* A. caviae* and *A. sobria* isolates into two groups. Single‐peak analysis revealed four discriminating peaks that separated *A. hydrophila* from *A. caviae* and *A. sobria* isolates. The resistance of *A. hydrophila* to antibiotics was 95.46% for ampicillin, 50% for cefotaxime, 45.45% for norfloxacin and pefloxacin, 36.36% for ceftazidime and ciprofloxacin, 31.81% for ofloxacin and 27.27% for nalidixic acid and tobramycin. In conclusion, chicken meat and water were tainted with *Aeromonas* spp., with a high occurrence of *A. hydrophila*. MALDI‐TOF MS is a powerful technique for characterizing aeromonads at the genus and species levels. Future studies should investigate the resistance of *A. hydrophila* to various antimicrobial agents.

## Graphical abstract

The sequencing results indicated that *A. hydrophila* is the most prevalent *Aeromonas* spp. isolated from food and water. MALDI‐TOF MS is a powerful technique used for identification of *Aeromonas* at the genus and species‐level. Principal component analysis (PCA) and single‐peak analysis are successful tools to discriminate the *Aeromonas* spp. VITEK^®^ 2 AST Cards can also use as a detective method of antimicrobial resistance.

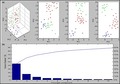

## 1 INTRODUCTION

Bacteria of the genus *Aeromonas* belong to the family Aeromonadaceae and include nineteen species (Aboyadak, Ali, Goda, Saad, & Salam, 2017; Demarta et al., 2008; Trakhna, Harf‐Monteil, Abdelnour, Maaroufi, & Gadonna‐Widehem, 2009) of gram‐negative, motile, nonlactose fermenting, nonspore forming, facultative anaerobic, and oxidase‐positive organisms. These bacteria can be classified into two large groups according to the host and physiological characteristics (Stratev & Odeyemi, 2016). The first group comprises motile aeromonads, represented by *Aeromonas hydrophila* (*A. hydrophila*), which causes various diseases mostly in mammals, including humans. The other group consists of nonmotile species, represented by *Aeromonas salmonicida*, which causes infections in fish (Bartkova, Kokotovic, Skall, Lorenzen, & Dalsgaard, 2017; Igbinosa, Igumbor, Aghdasi, Tom, & Okoh, 2012). Motile *Aeromonas* spp. are pathogens that cause foodborne gastroenteritis in humans and extraintestinal infections, such as bacteremia, soft tissue infections, meningitis, endocarditis and osteomyelitis (Alhazmi, 2015), with a high mortality rate in immunocompromised hosts (Gauthier, Vincent, Charette, & Derome, 2017; Igbinosa et al., 2012; Koca & Sarimehmetoglu, 2009; Steinberg & Burd, 2010).

Many people consume chickens daily as a source of animal protein worldwide; hence, hygienic methods of supplying chickens for consumption are critical for public health. Meat can be infected with *Aeromonas* spp. not only through inadequate processing, cutting and grinding but also by washing carcasses with contaminated water (Ghenghesh, Ahmed, El‐Khalek, Al‐Gendy, & Klena, 2008; Stratev & Odeyemi, 2016). The poor hygienic conditions associated with the processing of raw meat are considered one of the major causes of *Aeromonas* spp. contamination of meat products (Encinas, Gonzalez, Garcia‐Lopez, & Otero, 1999; Ogu, Madar, Okolo, & Tayubi, 2017; Rajakumar, Ayyasamm, Shanthi, Song, & Lak‐shmanaperumalsamy, 2012). Therefore, the genus* Aeromonas* has been associated with a wide variety of food and waterborne infections worldwide, particularly in less developed countries due to poor personal hygiene and lack of quality water (Odeyemi & Ahmad, 2017). Most *Aeromonas* spp. are virulent due to their ability to multiply and produce several toxins in refrigerated conditions (Eley, Geary, & Wilcox, 1993; Kirov, 1993; Humphries & Linscott, 2015; Miyagi, Hirai., & Sano, K., 2016). Because *Aeromonas* spp. represent commonly isolated pathogens from food as a result of their survival in water and human and animal feces, the threats of foodborne infections with *Aeromonas* are augmented (Ahmed, Abd El Aal, Ayoub, & Sayed, 2014; Koca & Sarimehmetoglu, 2009).

The most important *Aeromonas* spp. are *A. hydrophila*,* Aeromonas caviae* (*A. caviae*), and *Aeromonas veronii* biovar *sobria* (*A. veronii* bv.* sobria*). These organisms are pervasive in water and meat (Encinas et al., 1999; Osman, Aly, Kheader, & Mabrok, 2012; Sharma & Kumar, 2011; Trakhna et al., 2009). *A. hydrophila* represents the most virulent of these species and produces multifactorial virulence factors, including structural features related to adhesion, cell attack, and escape from the phagocytosis process, and certain extracellular factors, such as aerolysin, which leads to lysis and toxicity of the cells (Abrami, Fivaz, Glauser, Parton, & Goot, 1998; Chopra & Houston, 1999; Citterio & Biavasco, 2015). Nevertheless, some species of *Aeromonas* were isolated formerly from several food products, and the substantial role of foods of animal origin in the distribution of *Aeromonas* infections is unclear.

Although biochemical methods, 16S rRNA sequencing and housekeeping genes are considered the standard methods for detecting different *Aeromonas* spp., they are not widely used due to their cost, labor and time requirements (Chen et al., 2014; Morinaga et al., 2013; Soler et al., 2004; Trakhna et al., 2009). In addition, the exactness of these presently available methods is limited, and the precise and rapid identification of *Aeromonas* at the species level is still problematic (Benagli et al., 2012; Pérez‐Sancho et al., 2018). From this perspective, to increase the rate of *Aeromonas* identification at the species level, recent studies have confirmed and recommended that matrix‐assisted laser desorption ionization–time of flight mass spectrometry (MALDI‐TOF MS) as an alternative technique for bacterial identification due to its favorable rapid application (Donohue, Smallwood, Pfaller, Rodgers, & Shoemaker, 2006; Elbehiry, Al‐Dubaib, Marzouk, Osman, & Edrees, 2016; Murray, 2010). This technology is an up‐to‐date approach extensively applied for the identification and discrimination of various microorganisms at the genus and species levels on the basis of MALDI‐TOF mass spectra (Elbehiry et al., 2017; Sandrin, Goldstein, & Schumaker, 2013; Vávrová, Balážová, Sedláček, Tvrzová, & Šedo, 2015).

The development of antimicrobial resistance in various types of bacteria is another significant challenge worldwide (Chugh, 2008; Laith & Najiah, 2013; Li & Webster, 2018). Recently, the antibiotic resistance of *Aeromonas* spp. has increased because resistance was developed not only in clinical isolates but also in strains isolated from different sources of food products (Alcaide, Blasco, & Esteve, 2010). Throughout the last decade, the distribution of antimicrobial resistance among foodborne pathogens has developed, possibly due to the prolonged administration of medications in the livestock used for human consumption (Adebayo, Majolagbe, Ola, & Ogundiran, 2012; Deng et al., 2016). A previous study conducted by Saavedra et al. (2004) illustrated that the widespread use of various groups of beta‐lactam antibiotics as a method of prophylaxis and treatment of *A. hydrophila* in humans and animals is considered one of the main causes of the increasing *A. hydrophila* resistance to amoxicillin, carbenicillin, and ticarcillin. Furthermore, the existence of resistance genes on mobile elements, such as plasmids, transposons and integrons, assists their rapid spread among microorganisms (Romero, Feijoo, & Navarrete, 2012). Similarly, the data on the incidence of antibiotic resistance in *Aeromonas* spp., particularly in *A. hydrophila* recovered from chicken meat and water, are sparse. Based on these previously mentioned data, our study was designed to identify various *Aeromonas* spp. from chicken meat and water samples using MALDI‐TOF MS confirmed by SYBR Green real‐time (RT)‐PCR and microchannel fluidics electrophoresis assays and to study the antimicrobial resistance of *A. hydrophila* using Vitek 2 Compact AST cards.

## 2 MATERIALS AND METHODS

### 2.1 Sample collection

A total of 150 samples, including chicken meat (*n* = 75) and water (*n* = 75), were collected from three different sites (Buraidah, Unaizah, and Albukairyah) in the Al‐Qassim region, Saudi Arabia. The samples were collected five times at nearly monthly intervals (in April, May, June, August, and September 2017). Three hundred grams of each chicken meat sample collected from six randomly selected local retail shops, and supermarkets were placed in a separate sterilized plastic bag for the isolation process. One hundred milliliters of each water sample was collected randomly from private drinking water wells, houses, and retailers. The samples collected from houses and retailers were treated first with a sterile sodium thiosulphate solution (13.2 mg/L) to neutralize chlorine and stop its bactericidal action (Massa, Armuzzi, Tosques, Canganella, & Trovatelli, 1999). All samples were kept under ice‐cold conditions, and bacteriological investigations were carried out within 2 hr of collection. All meat and water samples were processed in the Microbiology Laboratory, College of Public Health and Health Informatics, Qassim University for isolation. Isolates were preserved in Cryobank vials at −80°C until the identification process was carried out.

### 2.2 Isolation of *Aeromonas* spp.

Thirty grams of each meat specimen was added to 225 ml of alkaline peptone water (pH 8.4 ± 0.2 at 25°C, Sigma‐Aldrich, USA), homogenized in a blender (Stomacher® 400, Thomas Scientific, USA) for 2 minutes and incubated at 30ºC for 18–24 hr. Likewise, 10 ml of each water sample was inoculated in 90 ml of peptone water with 1% NaCl (w/v) at pH 8.6 adjusted with sodium hydroxide and incubated at 30ºC for 18–24 hr. The cultures were streaked onto Aeromonas Isolation Agar (Sigma‐Aldrich) containing 5 mg/L ampicillin, which supports the growth of *Aeromonas* spp. After incubation of all plates at 28ºC for 24–48 hr, the colonies appeared slightly to deep green. Three to five typical colonies were subcultured onto glutamate starch phenol red agar (Sigma‐Aldrich), and after incubation, the colonies appeared as yellow colonies surrounded by a yellow zone and were identified primarily as *Aeromonas* spp. if they were gram‐negative, oxidase‐positive and glucose fermenting. The Voges–Proskauer reaction; esculin hydrolysis; lysine decarboxylase; and fermentation of arabinose, salicin, and sorbitol were then carried out to differentiate *Aeromonas* at the species level (*A. hydrophila*,* A. caviae,* and *A. veronii* bv.* sobria*) according to the method described by Janda, Abbott, and Carnahan (1995).

### 2.3 Biochemical analysis of *Aeromonas* using the Vitek 2 Compact system

The Vitek 2 Compact ((bioMérieux. Marcy l'Etoile, France) Gram‐Negative Identification (GNI) and antibiotic susceptibility testing (AST) cards were used to identify and determine the antibiotic susceptibilities of *Aeromonas* spp. according to the manufacturer's recommendations. In brief, 3–4 fresh colonies were suspended in sterilized physiological saline (aqueous 0.45% NaCl, pH 4.5 to 7.0) and thoroughly mixed. The Mcfarland turbidity was adjusted in the range from 0.50 to 0.63 using DensiChekTM (BioMe′rieux, France). Five milliliters of this suspension was loaded into Vitek 2 ID‐GNI and AST gram‐negative (AST‐GN04) cards. The Vitek 2 Cassette was finally loaded with cards and suspension tubes into the device. The unknown organisms were compared to the reference strains stored in the Vitek 2 Compact software for proper identification.

### 2.4 Rapid identification of *Aeromonas* spp. using MALDI Biotyper

We applied MALDI Biotyper Reference Library for Clinical Applications (MBT‐CA) Database version V.3.3.1.2 (Bruker Daltonik, Bremen, Germany) which has been approved by FDA under Section 510(k) as a powerful method for rapid and precise identification and discrimination of *Aeromonas* spp. The Proteomic identification was performed according to the ethanol/formic acid extraction method designated by Bruker Corporation. Briefly, a fresh colony of overnight culture, incubated at 28°C for 24 hr, was utilized for each isolate and inoculated onto two spots of the target plate, and every colony was then covered with 1 µl of matrix solution (saturated α‐cyano‐4‐hydroxycinnamic acid in 50% acetonitrile and 2.5% trifluoroacetic acid). The microbial spectra were directly produced by applying Compass IVD software, and the identification was directly conducted with a MALDI Biotyper machine.

### 2.5 Data analysis in MALDI Biotyper

The score value of the unidentified spectrum in the range from zero to three was determined by matching the unknown spectra with the spectra stored in the Bruker library. The accuracy of the strain recognition was detected as designated by the measures of Bruker Daltonik. The device performed the precise detection of species when the log score ranged from 2.3 to 3.0; nevertheless, the species and genus levels were recognized in the range from 2.00 to 2.29 and from 1,700 to 1,999, respectively. Furthermore, a score of 0.00 to 1.69 means that the proof of identity is not reliable. The diverse spectra created by the Microflex LT Compass IVD software were measured in a m/z range from 2,000 to 20,000 Da. To distinguish between *Aeromonas* spp., mathematical testing of the data sets was generated on the basis of principal component analysis (PCA), and the findings were illustrated in a three‐dimensional (3d) score plot created directly by compass software. According to the MBT‐CA Database, which contains 47 reference *Aeromonas* spp. and subspecies, the PCA dendrogram setting was utilized for species grouping.

### 2.6 Molecular identification of *Aeromonas* spp. using SYBR Green RT‐PCR

#### 2.6.1 DNA extraction

DNA extraction of the field isolates was achieved by QuickGene‐810 (AutoGen, Japan) using the QuickGene DNA tissue kit S (DT‐S), which was applied according to the manufacturer's recommendations. Briefly, 3–5 fresh colonies of each sample grown on soybean casein digest agar were transferred into a sterilized microcentrifuge tube containing 180 µl MDT lysis buffer and 20 µl proteinase K, and the lysate was then centrifuged at 8,000 g for 5 min. The supernatant was transferred to a new Eppendorf tube, and 180 µl LDT buffer was added. Two hundred forty microliters of absolute ethanol (Panreac, Barcelona, Spain) was added, and the tube was properly agitated. The lysate was transferred into the cartridge supplied with the kits and then inserted into the machine. Finally, the concentration and purity of the extracted DNA were determined by the NanoDrop™ 2000 spectrophotometer (Thermo Scientific, MA, USA).

#### 2.6.2 Primers used in the study

The isolates were further confirmed to the species level by *16S rRNA*, aerolysin (*aerA*), polar flagella (*Fla*), and hemolysin (*ASA1*) genes analysis. A specific *16S rRNA* region was carefully chosen for detecting *Aeromonas* spp. The primer express software, ver. 2.0 (Applied Biosystems, USA) was used to designate the primers, and their specificity was investigated with the BLAST program (Table 1).

**Table 1:** Oligonucleotide primers used to detect *A. hydrophila, A. caviae,* and *A. sobria* genes

Target gene	Oligonucleotide sequence (*5’−3’)*	GenBank accession number	Size (bp)
*16S rRNA*‐F	GGCCTTGCGCGATTGTATAT	DQ455052	103
*16S rRNA*‐R	GTGGCGGATCATCTTCTCAGA		
*AerA*‐F	CAAGGCTGATATCTCCTATCCCTATG	AF485770	67
*AerA*‐R	GCCACTCAGGGTCAGGTCAT	AY352352	
*Fla‐F*	TCCAACCGTYTGACCTC	AF198617	608
*Fla‐R*	GMYTGGTTGCGRATGGT	AF002709	
*ASA1‐F*	TAA AGG GAA ATA ATG ACG GCG	X65046	249
*ASA1‐R*	GGC TGT AGG TAT CGG TTT TCG		

#### 2.6.3 SYBR Green RT‐PCR assay and electrophoresis for PCR products

SYBR Green RT‐PCR for detection of the *A. hydrophila, A. caviae,* and *A. sobria* specific genes was then performed using a 7500 Fast Real‐Time PCR System (Applied Biosystems). Briefly, a 20 µl reaction volume containing 10 µl of Maxima SYBR Green qPCR Master Mix (2×), no ROX (Thermo Scientifics), 1 µl forward primer, 1 µl reverse primer, 1 µl target DNA and 7 µl of RNase/DNase free water was used. All reactions were carried out in duplicate. Regular amplification parameters were carried out as follows: 50°C for 2 min, 95°C for 2 min, followed by 40 amplification cycles, each of which comprised 95°C for 15 s and 60°C for 1 min. Amplification results were expressed by plotting Delta Rn (ΔRn) versus cycle number for detection of the *Aeromonas* genes. Electrophoresis for PCR products was then carried out using a LabChip GX Touch 24 device (PerkinElmer, USA). DNA 1 K Assay Quick was used for chip and preparation of samples according to the manufacturer's procedures.

### 2.7 Antimicrobial resistance of *A. hydrophila* using Vitek 2 Compact AST cards

Vitek 2 Compact AST‐GN04 cards (MedexSupply, Passaic, NJ, USA) were used to determine the susceptibility of *A. hydrophila* to antimicrobial agents. As shown in Table 5, five groups of antibiotics were tested in the present study. All antimicrobial agents were chosen according to these five groups, which can be measured by Vitek 2 system cards (Cockerill et al., 1995). Throughout the evaluation period, *A. hydrophila* ATCC 35654 was used as a quality control strain and was checked at regular intervals.

### 2.8 Statistical analysis

The data obtained from our study were imported into the Statistical Package for the Social Sciences (SPSS), and all estimations were carried out using SPSS version 20.0 (SPSS Inc., Chicago, IL, USA).

## 3 RESULTS

### 3.1 Incidence of *Aeromonas* spp. in chicken meat and water

The incidence of *Aeromonas* was examined in 75 chicken meat and another 75 water samples. According to our findings, of 150 chicken meat and water samples, 43 (28.66%) were positive for *Aeromonas* spp. Out of 75 chicken meat and 75 water samples, 31 (43.33%) and 12 (16%) were positive for *Aeromonas* spp., respectively. Among the positive samples, 22 (51.16%) *A. hydrophila*, 12 (27.9%) *A. caviae,* and 9 (20.93%) *A. sobria* strains were isolated from both chicken meat and water samples (Table 2). Of the 31 chicken meat samples recognized as tainted with *Aeromonas* spp., 17 (54.83%) *A. hydrophila*, 8 (25.8%) *A. caviae,* and 6 (19.35%) *A. sobria* strains were isolated. Of the 12 positive water samples for *Aeromonas* spp., 5 (41.66%), 4 (33.33%), and 3 (25%) were positive for *A. hydrophila*,* A. caviae,* and *A. sobria*, respectively (Table 2).

**Table 2:** Frequency of *Aeromonas* species in positive chicken meat and water samples

Sample origin	Positive samples		*Aeromonas* species					
			*A. hydrophila*		*A. caviae*		*A. sobria*	
	No.	%	No.	%	No.	%	No.	%
Chicken meat	31	72.1	17	54.83	8	25.80	6	19.35
Water	12	27.9	5	41.66	4	33.33	3	25
Total	43	100	22	51.16	12	27.90	9	20.93

### 3.2 Biochemical identification of *Aeromonas* isolates

The Vitek™ 2 Compact system properly identified 39 of 43 (90.69%) *Aeromonas* spp., as 21/22 (95.45%) strains of *A. hydrophila*, 10/12 (83.33%) strains of *A. caviae* and 8/9 (88.88%) strains of *A. sobria* (Table 3).

**Table 3:** Identification of *Aeromonas* spp. recovered from chicken meat and water using Vitek™ II Compact ID‐GNI cards

*Aeromonas* spp.	No. of tested isolates	Correctly identified		Misidentified	Not identified
		No.	%		
*A. hydrophila*	22	21	95.45	1	0
*A. caviae*	12	10	83.33	0	2
*A. sobria*	9	8	88.88	1	0
Total	43	39	90.69	2	2

### 3.3 Accurate identification of *Aeromonas* spp. using MALDI‐TOF MS

**Figure 1:**
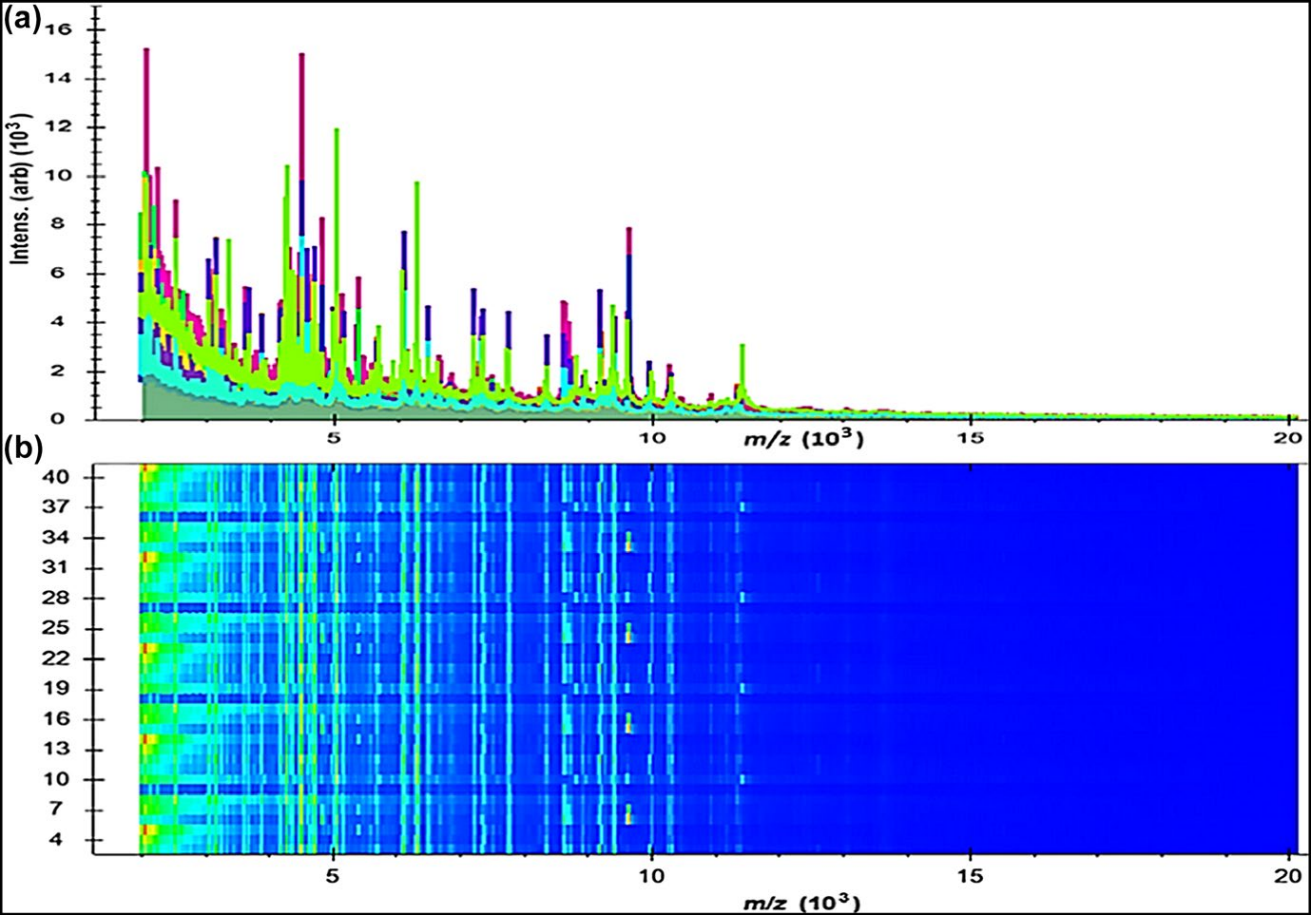
Mass spectrum protein profiles of 43 *Aeromonas* spp.; (a) Distribution of peaks within the line spectra ranging from 2,000 to 11,000 Da; (b) The gel profile of protein spectra in which the varied color of spots was the gathering of spectra with several contents

**Figure 2:**
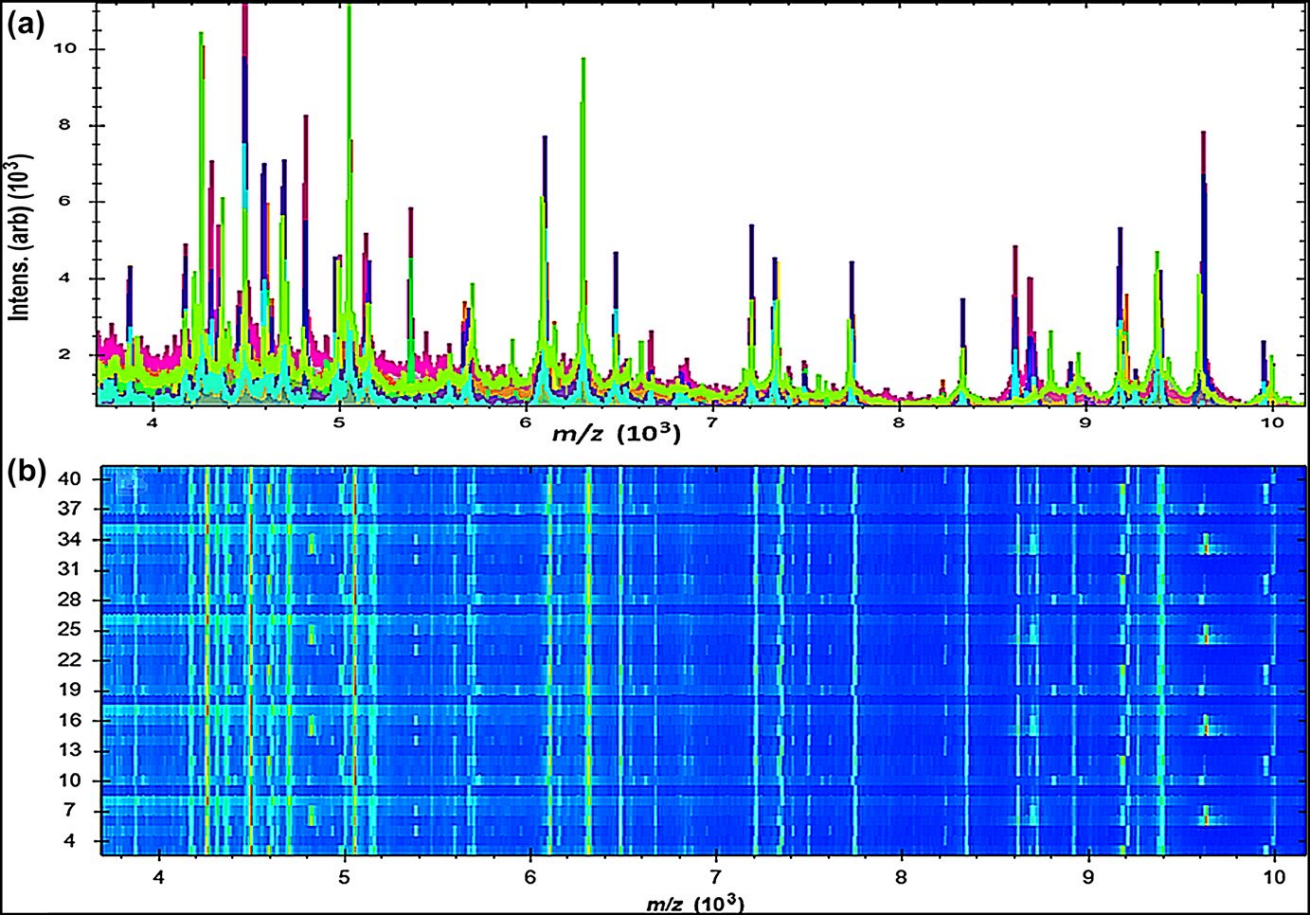
Mass spectral profiles of 43 *Aeromonas* spp.; (a) higher strength peaks were scattered within the line spectra ranging from 4,000 to 10,000 Da; (b) The gel profile of protein spectra distributed within the same range

In the current study, 43 *Aeromonas* isolates were investigated by the Microflex LT device, and the generated spectra were compared with the stored spectra in the Bruker library of Compass software. The precise identification rates for species listed in the Bruker Daltonics Compass 2.0 database by the MALDI Biotyper system were 21/22 (95.45%) for *A. hydrophila*, 12/12 (100%) for *A. caviae,* and 9/9 (100%) for *A. sobria*. In Table 4, we report that 9/22 (40.90%) *A. hydrophila*, 4/12 (33.33%) *A. caviae,* and 4/9 (44.44%) *A. sobria* were correctly identified at the species level, with a score value ranging from 2,300 to 3,000. Moreover, 12/22 (54.54.76%) *A. hydrophila*, 8/12 (66.66%) *A. caviae,* and 4/9 (44.44%) *A. sobria* were also identified at the species level, with a score value ranging from 2,000 to 2,299. However, one isolate of *A. hydrophila* was identified at the genus level with a score value ranging from 1.7 to 1.99. In contrast, zero isolates were not identified. A current gel view demonstrated the created spectra for all *Aeromonas* spp. Several spectra were distributed within the range from 2,000 to 11,000 m/z (Figure 1), and the higher peaks were determined between 4,000 and 10,000 m/z (Figure 2).

**Table 4:** Score values for 43 *Aeromonas* species of broiler chicken identified by MALDI Biotyper

Category	Score range	Identification level	*Aeromonas* species					
			*A. hydrophila*		*A. caviae*		*A. sobria*	
			No.	%	No.	%	No.	%
1	2.3–3	Species	9/22	40.90	4/12	33.33	4/9	44.44
2	2–2.29	Species	12/22	54.54	8/12	66.66	4/9	44.44
3	1.7–1.9	Genus	1/22	4.54	0/12	0	1/9	11.11
4	0–1.6	Not identified	0/22	0	0/12	0	0/9	0

**Figure 3:**
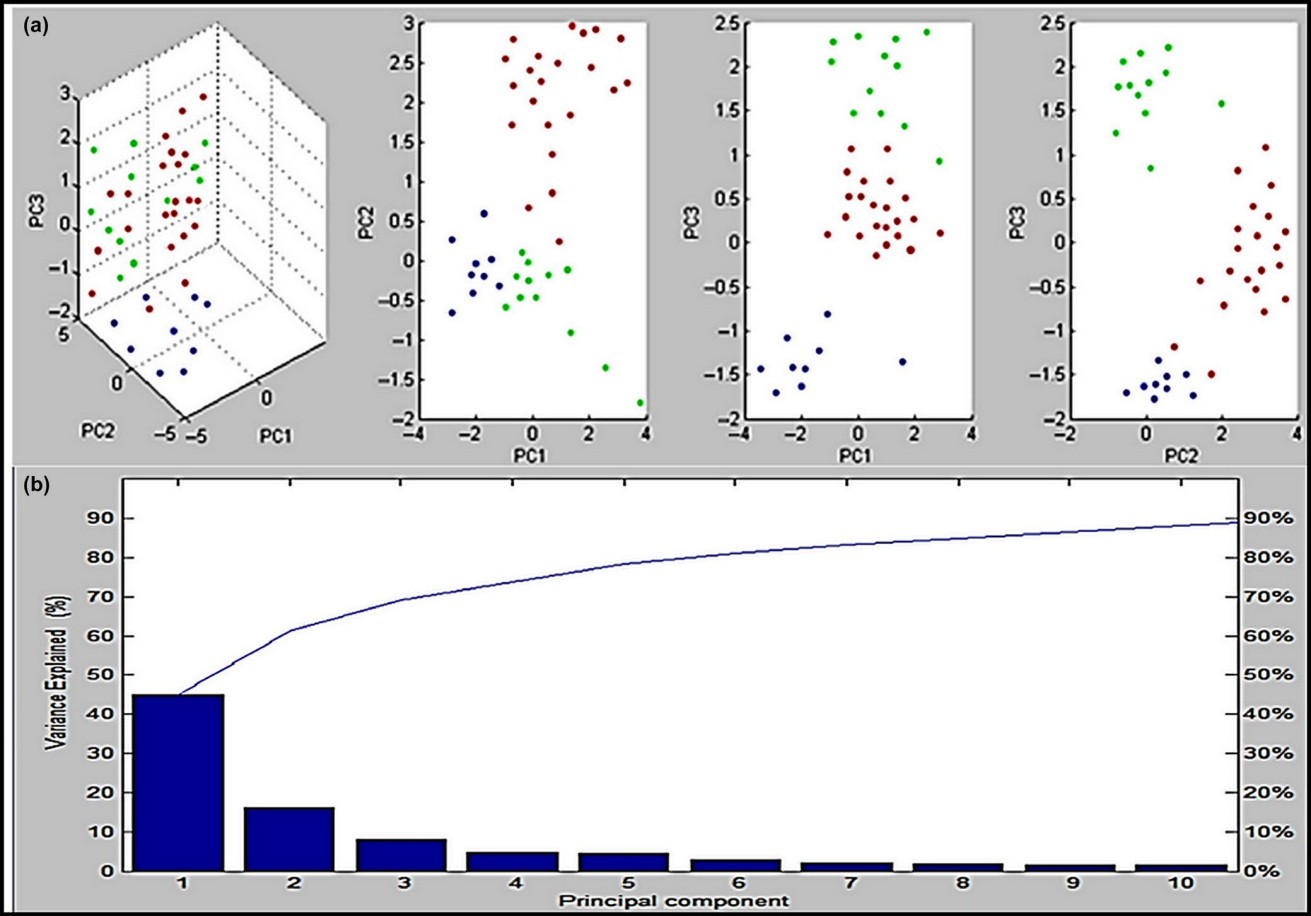
The dimensional image from PCA displays the difference between 43 *Aeromonas* spp.; (a) the grouping of *A. hydrophila* (red), *A. caviae* (green), and *A. sobria* (blue) in the first three model of PC (PC1, PC2, PC3); (b) the influence of ten principal components to the profiling classification in plot of percentage explained variance of PC. The contributions of PC1, PC2, and PC3 were around 45%, 17%, and 9%, correspondingly

Furthermore, a supplementary mathematical tool called PCA was generated in our study by MALDI Biotyper Compass software to explore the degree of similarity and variation in the protein spectra. Numerous protein spectra of the identified isolates were clarified in three‐dimensional (3d) PCA as shown in Figure 3a. Each peak was identified with 3 loading values originating from the calculation of three principal components (PC1, PC2, and PC3). In our analysis, the entire peaks listed in the MALDI Biotyper Compass 2.0 database were analyzed by the PCA tool, which separated *A. hydrophila*,* A. sobria,* and *A. caviae* isolates into two distinctive groups, as shown in the 3d PCA. Nevertheless, two strains of *A. hydrophila* were found in the *A. sobria* cluster. The *A. caviae* strains did not create a distinct group but were localized in the *A. hydrophila* cluster (Figure 3a). Based on the PCA calculation, the impacts of PC1, PC2, and PC3 on the creation of a profile in a percentage plot of the difference elucidated were nearly 45%, 17%, and 9%, respectively (Figure 3b).

**Figure 4:**
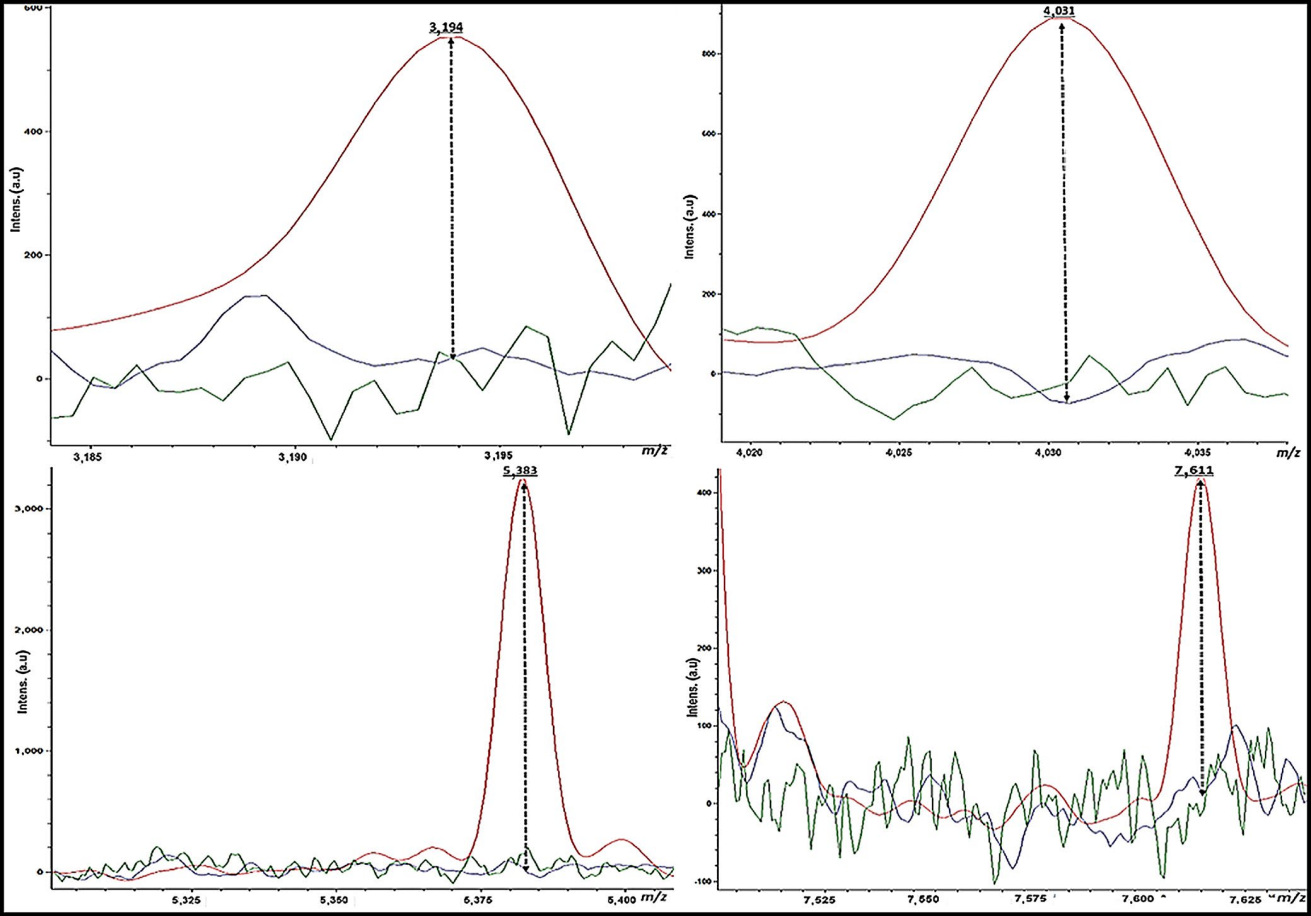
Higher peaks intensity (3,194, 4,030, 5,383, and 7,611 Da) were detected in *A. hydrophila* (red), whereas they were missed in *A. sobria* (green) and *A. caviae* (blue)

**Figure 5:**
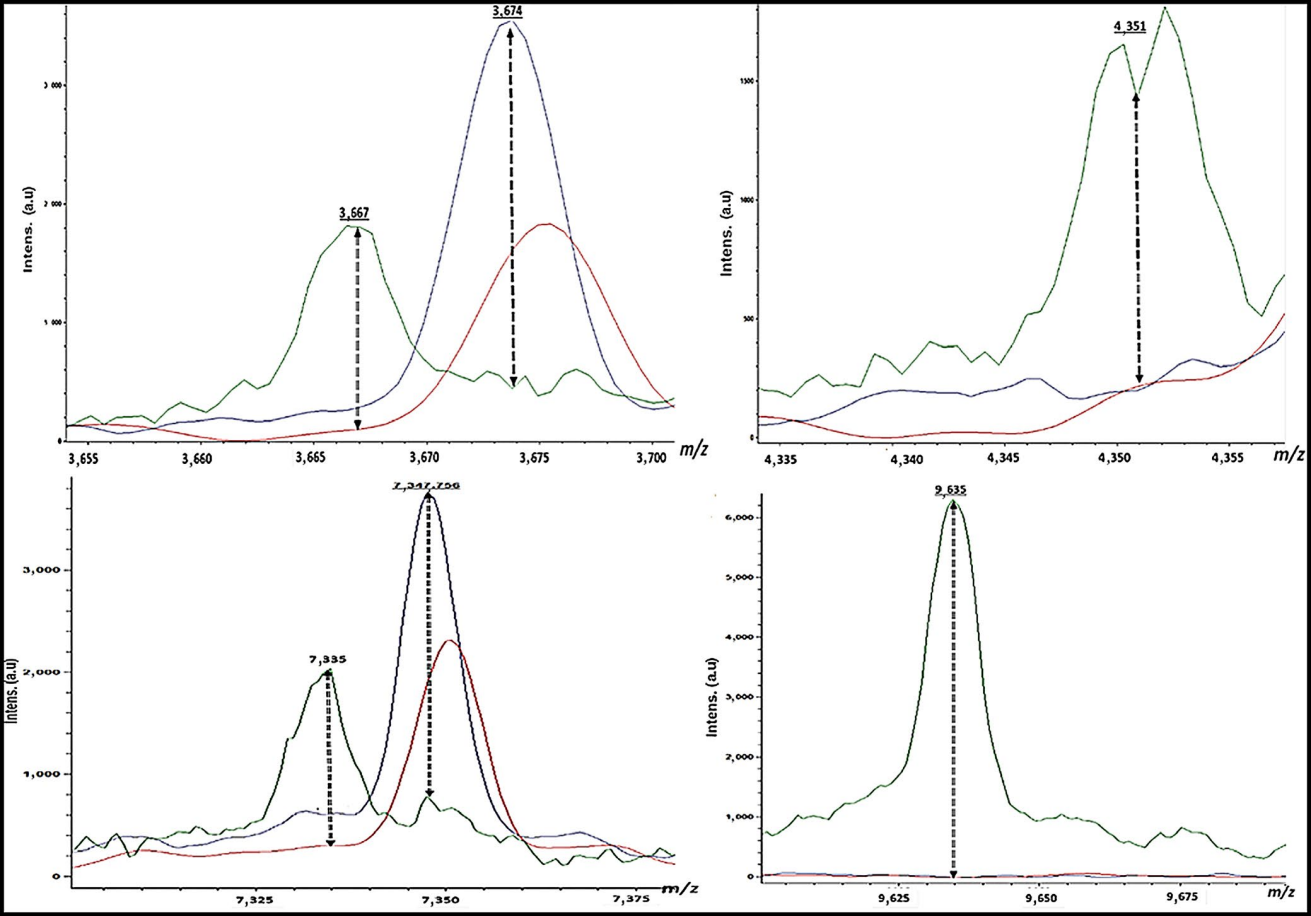
Higher peaks intensity (3,667, 4,351, 7,335 and 9,635 Da) were detected in *A. sobria* (green), whereas they were missed in *A. hydrophila* (red) and *A. caviae* (blue). Moreover, a higher peak intensity (7,347 Da) was detected in *A. caviae* (blue) while it was missed in *A. hydrophila* (red) and *A. sobria* (green)

Likewise, we analyzed a single peak for all *Aeromonas* spp. to explore the distinctive differences in the three *Aeromonas* spp. Higher peak intensities were detected in *A. hydrophila* at 3,194 Da, 4,031 Da, 5,383, and 7,611 m/z, whereas they were missed in *A. caviae* and *A. sobria* (Figure 4). Otherwise, the averaged spectra of *A. sobria* isolates exhibited definite peaks at 3,367, 4,351, 7,335, and 9,635 m/z, whereas they were missed in *A. hydrophila* and *A. caviae*. Moreover, the higher peak intensity at 7,347 m/z was identified in *A. caviae* and absent in both *A. sobria* and *A. hydrophila* (Figure 5). Analysis of *A. hydrophila* spectra demonstrated that the 3,194, 4,031, 5,383, and 7,611 m/z peaks were frequently found in 59% (13/22), 54.5% (12/22), 90.9% (20/22), and 95.45% (21/22) of the *A. hydrophila* strains, respectively. Moreover, the 3,367, 4,351, 7,335, and 9,635 m/z peaks commonly existed in ~67% (6/9), ~89% (8/9), ~78% (7/9), and 100% (9/9), of the *A. sobria* spectra, respectively.

### 3.4 Confirmation of the identification of *Aeromonas* isolates using SYBR Green RT‐PCR

The SYBR Green RT‐PCR technique was then carried out to confirm the MALDI Biotyper results. The primers precisely targeting regions of the *A. hydrophila*,* A. caviae,* and *A. sobria*,* 16S rRNA*,* aerA*,* fla,* and *ASA1* genes were designed to identify pathogenic *A. hydrophila, A. caviae and A. sobria* strains. PCR amplification with these primers yielded amplicons of the expected molecular weights. Amplification of each gene was tested separately, and the size of each expected product was confirmed. The sizes obtained after the LabChip analysis were 107, 67, 608 and 249 bp for the *16S rRNA*,* aerA*,* fla,* and *ASA1* PCR products, respectively. Comparing the results of identification accomplished by MALDI‐TOF Mass Spectrometry and SYBR Green RT‐PCR illustrated an agreement of 100%; therefore, the PCR was succeeded to confirm the results of MALDI.

### 3.5 Antimicrobial resistance of *A. hydrophila*

Vitek 2 Compact AST‐GN04 cards were used to determine the susceptibility of *A. hydrophila* to antimicrobial agents (Table 5). Our findings indicated that 51.16%, 27.90%, and 20.93% of bacterial isolates recovered from chicken meat and water samples were *A. hydrophila*,* A. caviae,* and *A. sobria*, respectively. As a result of these findings, we focused on *A. hydrophila* resistance to various antimicrobial agents using Vitek 2 Compact cards. As shown in Table 5, of 22 *A. hydrophila* isolates, 21 (95.46%) were resistant to ampicillin (beta‐lactam penicillins), but all strains were sensitive to piperacillin, ticarcillin and beta‐lactam/beta‐lactam inhibitors (amoxicillin/clavulanic acid, piperacillin/tazobactam, ticarcillin/clavulanic acid). A total of 50%, 36.36%, and 18.18% of *A. hydrophila* isolates were resistant to third‐generation cephalosporins (cefotaxime, ceftazidime, and cefpodoxime), respectively, whereas fourth‐generation cephalosporins (cefepime and cefpirome) showed strong activity against all tested isolates. A total of 31.81%, 45.45%, 45.45%, 27.27%, and 36.36% of *A. hydrophila* isolates were resistant to the tested quinolones (ofloxacin, norfloxacin, pefloxacin, nalidixic acid, and ciprofloxacin), respectively. In contrast, the susceptibility of *A. hydrophila* to aminoglycosides was 100% for netilmicin and isepamicin, 95.45% for amikacin and gentamicin and 63.63% for tobramycin.

**Table 5:** Susceptibility percentage for 22 *A. hydrophila* strains recovered from chicken meat and water samples

Antimicrobial agent	Conc. (µg)	Vitek 2 Compact system					
		S		I		R	
		No	%	No	%	No	%
Beta‐lactam penicillins							
Ampicillin	10	1	4.54	0	0	21	95.46
Piperacillin	100	22	100	0	0	0	0
Ticarcillin	75	22	100	0	0	0	0
Beta‐lactam/Beta‐lactam inhibitors							
Amoxicillin/clavulanic acid	20/10	22	100	0	0	0	0
Piperacillin/tazobactam	100/20	22	100	0	0	0	0
Ticarcillin/clavulanic acid	75/10	22	100	0	0	0	0
Cephalosporins							
Cefotaxime	30	11	50.00	0	0	11	50.00
Ceftazidime	30	14	63.63	0	0	8	36.36
Cefpdoxime	10	17	77.27	1	4.54	4	18.18
Cefepime	30	22	100	0	0	0	0
Cefpirome	30	22	100	0	0	0	0
Quinolones							
Ofloxacin	5	11	50.00	4	18.18	7	31.81
Norfloxacin	10	12	54.54	0	0	10	45.45
Pefloxacin	30	12	54.54	0	0	10	45.45
Nalidixic acid	30	12	54.54	1	4.54	9	27.27
Ciprofloxacin	10	13	59.10	3	13.63	6	36.36
Aminoglycosides							
Amikacin	30	21	95.45	0	0	0	4.55
Gentamicin	10	21	95.45	0	0	0	4.55
Netilmicin	30	22	100	0	0	0	0
Isepamicin	30	22	100	0	0	0	0
Tobramycin	10	14	63.63	2	9.10	6	27.27

## 4 DISCUSSION

The accurate identification of various pathogens is an essential step of diagnosis, and the time‐to‐result obtained is very significant to start the selected treatment as soon as possible. Rapid and accurate analytical tools are necessary for monitoring the food and water safety and screening of any undesirable pathogens, which may cause noteworthy health hazards upon consumption. *Aeromonas* spp. are known to cause various infections in humans. Because its developing significance as an emerging pathogen isolated from food and water, it is imperative to combat this bacterium (Praveen, Debnath, Shekhar, Dalai, & Ganguly, 2016).

As culture‐ and biochemical‐based identification of different microorganisms are difficult and time‐consuming, MALDI‐TOF MS was significantly used here for the early identification and discrimination of various pathogens from environmental samples by introducing a simple, rapid, precise, and low‐cost identification method compared to other methods (Elbehiry et al., 2016; Singhal, Kumar, Kanaujia, & Virdi, 2015; van Belkum, Welker, Pincus, Charrier, & Girard, 2017). Recently, MALDI‐TOF MS has been revealed to be an important method for the rapid identification of bacterial threats that might contaminate drinking water and food products (Singhal et al., 2015).

In our study, the identification rates for *Aeromonas* isolates were 21/22 (95.45%) for *A. hydrophila*, 12/12 (100%) for *A. caviae* and 9/9 (100%) for *A. sobria*. These findings prove that the mass spectral data generated by MALDI Biotyper Compass 2.0 Software for all isolates were satisfactory to differentiate between the genus *Aeromonas* at the species and strain levels. The higher level of precise identification compared to that of the former studies might be due to the updated Compass 2.0 database utilized in our study (Lo et al., 2015; Seng et al., 2009). Similar results were obtained by Donohue et al. (2007), who used the *m*/*z* signature of the recognized *Aeromonas* reference isolates to allocate species of unidentified environmental isolates. They reported that MALDI‐TOF MS quickly and precisely categorized fourteen species and four subspecies of *Aeromonas*, including *A. hydrophila*,* A. caviae*,* A. jandaei,* and *A. veronii* bv*. sobria*, which were the most clinically significant species of the genus *Aeromonas*.

A previous study of *Aeromonas* isolates was also conducted by Donohue et al. (2006), who indicated that the signals created by MALDI‐TOF MS Compass 2.0 Software after analysis of protein spectra might be utilized as specific biomarkers for the successful identification and discrimination of *Aeromonas* at the species and below the species level. Another study was carried out by Chen et al. (2014) for proteomic identification of 217 *Aeromonas* strains using cluster analysis of spectra created by MALDI‐TOF MS. They reported that the *Aeromonas* strains were precisely identified as 96.7% *S* (*A. dhakensis*), 90% *A. hydrophila*, 96.7% *A. veronii*, and 100% *A. caviae*. Böhme et al. (2011) utilized MALDI‐TOF MS successfully in the accurate identification of 26 species of seafood spoilage and pathogenic gram‐negative bacteria, including *A. hydrophila*,* Acinetobacter baumannii*,* Pseudomonas* spp., and *Enterobacter* spp. Therefore, proteomic identification has been illustrated to be a powerful tool for species identification. Another MS study was evaluated by Lamy, Kodjo, Laurent, and CoIBVH Group (2011) for proteomic identification of aeromonads. They found that the genus‐level precision was detected at 100% compared with *rpoB* gene sequencing, which makes this system one of the most reliable and rapid techniques for the identification of various microorganisms.

The main benefit of MALDI‐TOF technology for routine diagnosis is the precise identification of various pathogens that, by traditional techniques, are frequently categorized to the genus or even genus‐group level (Elbehiry et al., 2017; García, Allende, Legarraga, Huilcaman, & Solari, 2012; McElvania TeKippe & Burnham, 2014; Porte et al., 2017). With MALDI‐TOF MS, these microorganisms have recently been identified without the high costs and significant time span related to multiple biochemical tests and/or 16S rRNA analysis (McElvania TeKippe & Burnham, 2014). Our assessment established that the MALDI‐TOF technology quickly and exactly identified nearly all *Aeromonas* isolates tested at the species level with a score value ≥2.00. However, one isolate of *A. hydrophila* in our study was identified at the genus level with a score value ranging from 1.7 to 1.99. Species identification by MALDI‐TOF MS is still not always reached as a result of small quantities of material, weak protein signals, and inadequate representation in the stored Compass 2.0 software (Bizzini et al.., 2011; Carrasco et al., 2016; Croxatto, Prod´hom, G., & Greub, G., 2012; Lau et al., 2014). Similar results were obtained by Benagli et al. (2012), who tested 741 clinical and environmental *Aeromonas* isolates using MALDI‐TOF MS and found that 93% of these strains were positively identified with a score value ≥2.00. In addition, we used MALDI‐TOF MS to discriminate between *A. hydrophila*,* A. caviae,* and *A. sobria* using PCA analysis and single‐peak analysis. PCA analysis successfully separated *A. hydrophila*,* A. caviae,* and *A. sobria* isolates into two groups. Single‐peak analysis revealed four discriminating peaks that separated *A. hydrophila* from *A. sobria* and *A. caviae* isolates. Han (2010) indicated that PCA is a commonly utilized calculation tool to extract, show and rank the difference within a data set. The main aim of PCA is to decrease the dimensionality of a data set, concurrently recollecting the information present in the data (Shao et al., 2012).

Likewise, the SYBR Green RT‐PCR established here was effectively used to confirm the identification of *A. hydrophila*,* A. caviae,* and *A. sobria* from chicken meat and water samples. For *16S rRNA*,* aerA*,* fla,* and *ASA1* gene detection, a good correlation between MALDI‐TOF MS and PCR analysis was found regardless of the origin of the isolates (meat or water). In addition to the LabChip preparation time (20 min), the PCR product can be shown with a microchannel fluidic apparatus in five min. After protein analysis, a molecular technique was used to identify the unconfirmed *Aeromonas* isolates in approximately 2.5 hr (Persson, Al‐Shuweli, Yapici, Jensen, & Olsen, 2015; Trakhna et al., 2009).

The distribution of drug resistance among *A. hydrophila* was also evaluated in our study, as previous surveys showed the development of this pathogen as one of the major opportunistic human pathogens (Laith & Najiah, 2013; Rey et al., 2009). The Vitek 2 Compact GN cards were used in the current study to detect the degree of resistance for 22 *A. hydrophila* against various antimicrobial agents commonly used for gram‐negative bacteria*.* Out of the 22 *A. hydrophila* isolates, 17 isolates were obtained from 75 chicken meat samples and 5 isolates were isolated from the 75 water samples. There was no significant difference between source of isolates in relation to their susceptibilities to various antibiotics.

Our findings revealed that 95.46% of *A. hydrophila* demonstrated a strong resistance to ampicillin among all the tested beta‐lactam penicillins. This finding was similar to previous studies conducted by Ramalivhana, Obi, and Moyo (2009) and Laith and Najiah (2013), who evaluated the susceptibility of different antimicrobial agents against *A. hydrophila* recovered from water and stool samples. They reported that 100% of isolates were resistant to ampicillin. Moreover, previous studies reported 100% *Aeromonas* resistance rates to ampicillin (Aoki, Egusa, Ogata, & Watanabe, 1971; Igbinosa, 2014; Rall et al., 1998). Most *A. hydrophila* isolates have intrinsic or chromosomally mediated resistance to ampicillin (Ghenghesh, El‐Mohammady, Levin, Zorgani, & Tawil, 2013; Rall et al., 1998).

In contrast, of the total *A. hydrophila* isolates tested in the current study, no resistance was detected against beta‐lactam/beta‐lactam inhibitors (amoxicillin/clavulanic acid, piperacillin/tazobactam and ticarcillin/clavulanic acid). A previous study was carried out by Awan, Maqbool, Bari, and Krovacek (2009), who evaluated the activity of ß‐lactam antibiotics against 20 *A. hydrophila* strains. They reported that *A. hydrophila* showed a high degree of resistance to ampicillin and cephaloridine, with the highest susceptibility to amoxicillin/clavulanic acid. However, some isolates were sensitive to third‐generation cephalosporins. As reported by Stratev and Odeyemi (2016), most *A. hydrophila* strains isolated from meat and meat products are resistant to a broad range of antimicrobial drugs.

Fourth‐generation cephalosporins, including cefepime and cefpirome, exhibited higher activity against all strains compared to third‐generation cephalosporins (cefotaxime, ceftazidime, and cefpodoxime), although some isolates were sensitive to these third‐generation cephalosporins, indicating that *A. hydrophila* has a variable susceptibility against cephalosporins. Similar results were obtained by Morita, Watanabe, Kurata, and Kanamori (1994) and Igbinosa (2014). Moreover, we observed that the aminoglycosides (gentamicin, amikacin, netilmicin, and isepamicin) showed excellent activity against all *A. hydrophila* strains. A similar report was observed by Awan et al. (2009), Dallal, Yazdi, and Avadisians (2012) and Igbinosa (2014), who found that *Aeromonas* spp. recovered from various food samples revealed sensitivity to gentamicin.

According to Alcaide et al. (2010), the resistance of *Aeromonas* spp. to various antibiotics has been augmented because emerging resistance has been established not only in clinical isolates but also in *Aeromonas* spp. recovered from water and food. Another study described by Adebayo et al. (2012) revealed that the frequency of bacterial resistance to different antibiotics in food and food products has increased throughout the last few years, potentially due to their extensive use in livestock raised for human feeding.

## 5 CONCLUSIONS

Current research illustrates a high frequency of possibly virulent *A. hydrophila* among *Aeromonas* spp. in chicken meat and water samples. Through this study, we confirmed that MALDI‐TOF MS used for the identification of *Aeromonas* isolates is a powerful, cost‐effective, and accurate method and was able to distinguish *A. hydrophila*,* A. caviae,* and *A. sobria* strains based on PCA and single‐peak analysis. RT‐PCR and microchannel fluidics electrophoresis assays can be used as a confirmatory diagnostic method for MALDI‐TOF MS. Future studies will address the application of this method for the direct identification and differentiation of *Aeromonas* spp. in food or water samples. Moreover, our findings demonstrate that the *A. hydrophila* strains in the sample had developed antibiotic resistance. Consequently, the progression of resistance may be predictable; accordingly, the number of effective antibiotics is declining. Because *A. hydrophila* may threaten human health, the transmission of resistance may have bad impacts for humans.

## CONFLICT OF INTEREST

The authors declare that they have no competing interests.

## AUTHORS CONTRIBUTION

AE, EM, and EEA designed the study. AE, EM, EEA, AA, and MH performed all the experiments, and AE, MA and EM analyzed the data and wrote the manuscript. All authors read and approved the final manuscript.

## ETHICS STATEMENT

Permission has been received from the owners of shops and retailers.

## DATA ACCESSIBILITY

All data generated or analyzed during this study are included in this published article.
